# Addressing infection in totally implantable venous access ports: An ex vivo study on bacterial transfer and the Forsvall Port needle design

**DOI:** 10.1177/11297298251383741

**Published:** 2025-12-07

**Authors:** Håkan Pärsson, Magnus Wagenius, Maria Utter, Jonas Tverring, José Francisco Pereira Cardoso, Andreas Forsvall

**Affiliations:** 1 Department of Health, Medicine and Caring Sciences, Linköping University, Linköping, Sweden; 2 Department of Surgery, Kalmar Hospital, Kalmar, Sweden; 3 Faculty of Medicine, Department of Clinical Sciences, Infection Medicine, Lund University, Lund, Sweden; 4 Department of Urology, Helsingborg Hospital, Helsingborg, Sweden; 5 Department of Infectious Diseases, Helsingborg Hospital, Helsingborg, Sweden; 6 Faculty of Medicine, Department of Dermatology and Venereology, Lund University, Lund, Sweden

**Keywords:** TIVAP, subcutaneous venous port, Huber needle, Forsvall Port needle, bacterial transfer, infection prevention, ex vivo study, catheter-related blood stream infection, CRBSI

## Abstract

**Background:**

Totally implantable venous access ports (TIVAPs) are essential for administering chemotherapeutic drugs and nutritional support, but carry a risk of infectious complications. We hypothesized that the design of the current port needle (Huber) could facilitate the collection of bacteria from the skin during puncture, potentially introducing them into TIVAPs and causing infections. This study examines bacterial transfer via needles during TIVAP access and proposes a novel needle design to reduce the risk of infection.

**Methods:**

A novel needle, the Forsvall Port needle, was developed with a closed tip to reduce bacterial transmission inside the needle during tissue and port penetration. In a randomized ex vivo setting, human skin samples covered in physiological levels of *Staphylococcus aureus* were placed over port membranes and punctured repeatedly by the standard Huber needle and the Forsvall Port needle. Cultures from the needle tips were plated after puncture and used to compare bacterial transfer into TIVAPs using a generalized linear mixed-effects model. Punctures were performed in a separate human skin to examine the mechanism of bacterial transfer.

**Results:**

The Forsvall Port needle reduced average bacterial transfer by 87.0% (95% CI: 77.8%–92.4%, *p* < 0.0001) compared with the Huber needle, based on 10 punctures per needle across four skin samples. Additional testing on a separate skin sample showed that a defectively fitted Forsvall Port needle prototype did not reduce bacterial transfer relative to the Huber needle (95% CI: 58.3% decrease to 30.0% increase, *p* = 0.289), whereas a steel rod simulating a perfectly closed Forsvall Port needle achieved a 99.9% (95% CI: 99.5%–100%, *p* < 0.0001) reduction in bacterial transfer.

**Conclusion:**

The Forsvall Port needle significantly reduces bacterial transfer into TIVAPs, which could potentially decrease TIVAP-related infections. This study demonstrates a strong association between needle design and bacterial transfer during TIVAP access.

## Introduction

Central long-term venous access is frequently required for the administration of chemotherapeutic drugs and nutritional support.^1^ To this end, totally implantable venous access ports (TIVAPs) have been in use since the early 1980s and are indicated when treatment duration exceeds 3 months.^2^ The implantation procedure typically involves a surgical cutdown or percutaneous access through the external jugular, internal jugular, or subclavian veins. Acute complications, such as bleeding and pneumothorax, are rare if proper techniques are employed.^3^ However, late TIVAP-related complications (onset >30 days from TIVAP implantation), including infections, thrombosis, catheter dislocation, and skin ulceration, have been reported in several studies.^3–5^

Late TIVAP infectious complication rates are significantly higher than other catheter-related and TIVAP-related issues,^5^ ranging from around 5% to as high as 13% despite preventive measures.^6–9^ These infections pose major clinical challenges, including the risk of septicemia and the need for TIVAP removal.^7^ While the source of infecting organisms is not entirely clear, infections most frequently involve skin microbes, such as *Staphylococcus aureus*,^7,10^ suggesting that TIVAP handling and the patient’s skin are significant sources of infection.^11^ Furthermore, an increased frequency of Huber needle punctures has been associated with a higher risk of bacterial transfer to the TIVAP and subsequent colonization.^12^

We have previously reported that bacterial translocation across the colon wall may be facilitated by bacterial accumulation between needle parts during prostate biopsy.^13^ In this case, bacterial transfer across colonic tissue was reduced by 96% using a redesigned smooth needle with a closed tip that prevented bacterial collection within the needle. These data demonstrated the significance of needle design in potentiating infections.

Medications and nutrients are delivered to TIVAPs using an open-tip Huber needle. Patented in 1946, the Huber needle was adapted for TIVAP use in the 1980s due to its non-coring design, which preserves the integrity of the TIVAP membrane during repeated punctures.^14^ However, based on its open-tip design, we hypothesized that bacteria from the skin surface and crypts^15^ could be collected inside the Huber needle during skin puncture and introduced into TIVAPs, leading to infections.

Using an ex vivo puncture model, this study aimed to evaluate the mechanism of bacterial transfer and assess whether a novel, smooth, closed-tip needle could reduce bacterial transfer through human skin into TIVAPs.

## Methods

### Needles and steel rod

A novel closed-tip needle prototype aiming to reduce bacterial translocation inside the needle was designed by Andreas Forsvall for use with TIVAPs. The novel 20G prototype, named the Forsvall Port needle after its inventor, features a two-part design with a closed tip that creates a smooth outer surface, drawing inspiration from the previously tested Forsvall Biopsy needle.^13^ The top of the Forsvall Port needle rotates 90°, allowing it to switch between a closed position to block bacterial entry during insertion and an open position for fluid transfer during use. It was designed to share the non-coring properties of the Huber needle.

**Figure 1.**
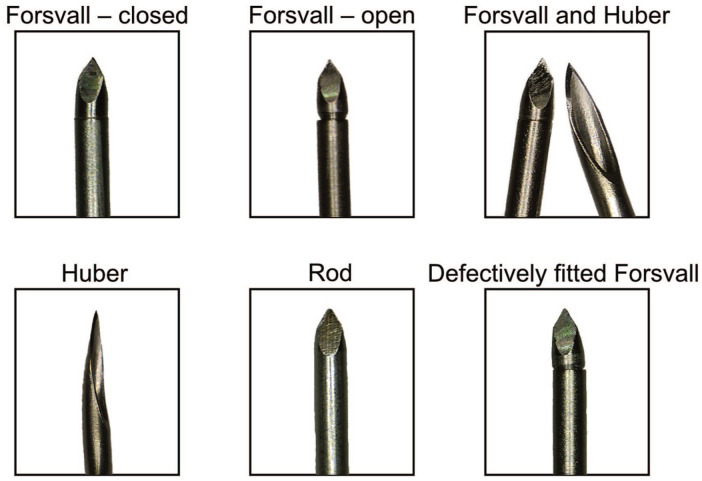
Microscopic pictures of needles used in this study. The needles are 0.9 mm in diameter. The background of the images was removed to enhance clarity.

A steel rod matching the size, material, and tip grinding of the Forsvall Port needle, but without separate parts or openings for bacterial collection, was developed to simulate a Forsvall Port needle with an optimal fit between its components. The Forsvall Port needle prototypes and steel rod that were used in this study were made from medical-grade stainless steel 304. The Huber needle was used as a reference (Surecan Safety II 20G, 32 mm; BBraun, Melsungen, Germany). Pictures of these needles can be found in Figure 1.

### Patient material

The study was approved by the Swedish Ethical Review Authority (Dnr: 2020-03956), and informed consent was obtained from all patients before surgery. Five human skin samples obtained from patients undergoing colostomy (abdominal skin) or breast reduction surgery between June 2022 and March 2024 at Helsingborg Hospital or Ängelholm Hospital were used. Inclusion criteria were patient consent and healthy, intact skin, while exclusion criteria were known skin diseases, birthmarks, or cortisone treatment (Table 1).

**Table 1.** Baseline characteristics of patients and skin specimens.

Patient	1	2	3	4	5
Tissue	Abdominal	Breast	Breast	Breast	Breast
Gender	M	F	F	F	F
Age (years)	67	55	40	70	73
Surgery	Colostomy	Breast reduction	Breast reduction	Breast reduction	Breast reduction
Needle study	Main^a^	Main	Main	Main	Substudy^b^

a The main study compared the Huber needle (currently used in clinical practice) and the Forsvall Port needle.

b The substudy compared the Huber needle, a defectively fitted version of the Forsvall Port needle, and a steel rod that stimulated a perfect fitting between the Forsvall Port needle parts.

### Bacterial culture

*Staphylococcus aureus* was used to quantify the transfer of a skin commensal onto agar plates, as the Newman strain (Genbank ID: AP009351.1)^16^ forms distinct colonies, with each colony representing a single transferred bacterium. *S. aureus* was inoculated into 7–8 mL of prewarmed Todd Hewitt (TH) broth. The culture was incubated overnight at 37°C with shaking, with the tube lid unsecured to allow for aeration. The following day, 1 mL of the overnight culture was transferred into tubes containing 6–7 mL of fresh, prewarmed TH media. The optical density (OD) at 620 nm was measured to determine the initial bacterial concentration. Bacteria were then cultured for an additional 1–2 h at 37°C with shaking and a loose lid, until reaching an OD of 0.4 at 620 nm (2 × 10^8^ colony-forming units (CFU)/mL). Bacterial cells were pelleted by 10-min centrifugation at 3000×*g*. The bacterial pellet was resuspended in sterile phosphate-buffered saline (PBS) to a concentration of 2 × 10^8^ CFU/mL and used at room temperature in experiments.

### Experimental design

**Figure 2.**
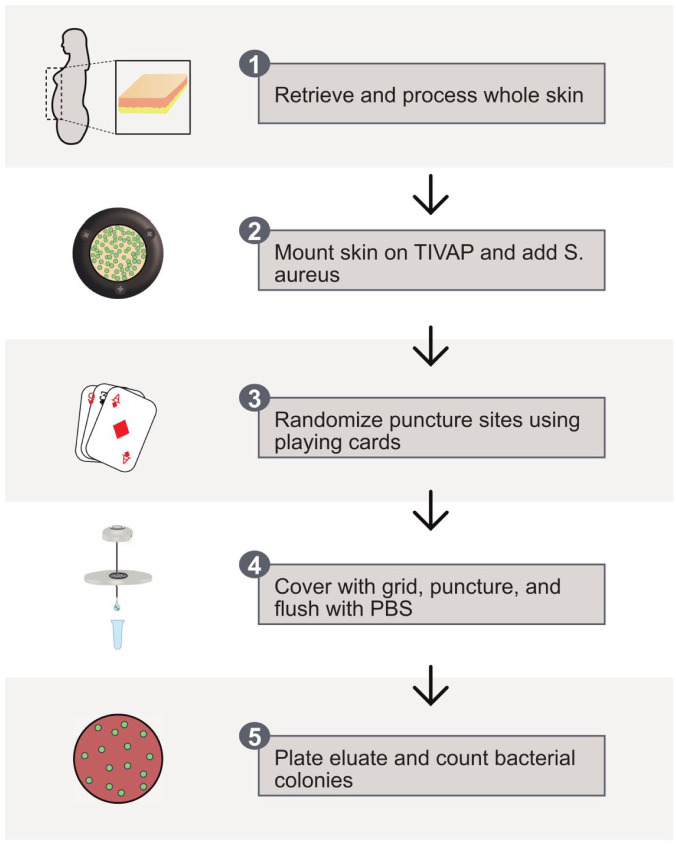
Schematic of the ex vivo skin puncture model used in this study.

**Figure 3.**
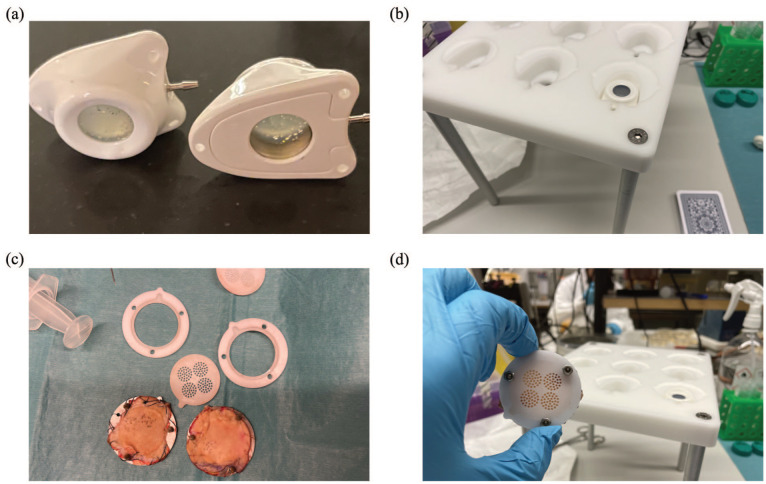
Bacterial translocation through ex vivo human skin following punctures with Huber and Forsvall Port needles. (a) The bottoms were removed from TIVAPs to allow for bacteria collection from the needles. (b) TIVAPs with the bottom removed were mounted in a holder. (c) Patient skin was mounted on a metal ring and bacteria were added. (d) A randomization grid was mounted over the skin prior to needle puncture. The skin was then mounted on top of the TIVAP.

A schematic of the experimental setup can be found in Figure 2. TIVAPs (Celsite T301P, BBraun, Melsungen, Germany) were modified by removing the bottom to allow for bacterial collection from the needles (Figure 3(a)). Before use, TIVAPs were cleaned, sterilized, and mounted on a custom-made bracket (Figure 3(b)). Immediately after removal, whole patient skin was prepared in a sterile environment by removing subcutaneous fat; the remaining skin was secured to a metal ring with continuous sutures along the edges (Figure 3(c)). Skin samples were smeared with 10^6^ CFU per cm^2^ with a plating loop and allowed to dry to simulate physiological skin bacteria concentrations presterilization. The test tube with bacteria was kept on ice until 15 min prior to usage. Given that needles penetrate all skin layers, we estimated the bacterial concentration based on a 2008 study reporting approximately 10⁶ bacteria per cm² in whole-skin punch biopsies.^17^ A randomization plate was placed on top of the skin to ensure consistent sampling (Figure 3(d)). The metal ring with skin was mounted over a TIVAP, replicating the clinical setup. All materials used were sterile.

### Randomization and skin puncture

Playing cards labeled with coordinates corresponding to holes in the randomization plate were shuffled for 5 min. The top card was drawn prior to each puncture to determine the puncture site. The needle was passed through the randomization plate, skin, and TIVAP membrane into a microtube, where 500 µL of PBS was injected and stirred with the needle for 3 s. The needle was then removed, sterilized in ethanol, rinsed twice in sterile water, and dried with a sterile cloth. Needle types were alternated between punctures in each skin sample. A new needle of each type was used for each skin sample. The Forsvall Port needle punctured the skin and TIVAP membrane in a closed state before opening to release its contents (Figure 1). In experiments using the steel rod, punctures were performed similarly to those with the needles, except the collection tubes were prefilled with 500 µL of PBS. After the rods punctured the skin and TIVAP, any bacteria collected on their surfaces were removed by stirring for 5 s. All procedures were completed within 3 h postsurgery to ensure skin viability.^18^

Volumes of 100 and 200 µL of the PBS from each puncture sample were spread on coded blood agar plates using a plating loop and incubated overnight at 37°C. CFUs were counted by an observer blinded to the study’s coding system. Each colony was considered to represent a single transferred bacterium.

### Statistical analysis and data presentation

The sample size in this study was adopted from the previously published study exploring bacterial transfer by the Forsvall needle across ex vivo colon tissue, which was conducted with 10 punctures in four colon tissue samples and demonstrated significant results with an acceptable margin.^13^ We expected to see similar levels of bacterial translocation and a similar effect size in this study.

To compare bacterial growth between the needles, we used a generalized linear mixed-effects model to account for dependencies in the data from the repeated measures within the skin punctures by using a random intercept. We used a negative binomial distribution since the bacterial growth was not normally distributed but showed a count data dispersion. Normality of residuals was assessed using quantile-quantile (QQ)-normal plots and histograms. Results are reported as percent reductions with 95% confidence intervals (95% CIs); *p*-values below 0.05 were regarded as statistically significant. Statistical analyses were conducted using Stata (Stata MP 18.0, StataCorp, Texas, USA). Graphs were created using GraphPad Prism (version 10.3.1, GraphPad Software, Massachusetts, USA).

## Results

### Design of the Forsvall Port needle

To design a closed-tip needle for use with TIVAPs, the existing TIVAP (Huber) needle was modified by integrating the two-part smooth surface design of the previously tested Forsvall Biopsy needle (Figure 1).^13^ The resulting Forsvall Port needle aims to maintain the non-coring and flow characteristics of the Huber needle but has a closed tip, designed to avoid accumulating bacteria inside the needle during skin puncture.

### Reduction of bacterial transfer through ex vivo human skin by the Forsvall Port needle

**Figure 4.**
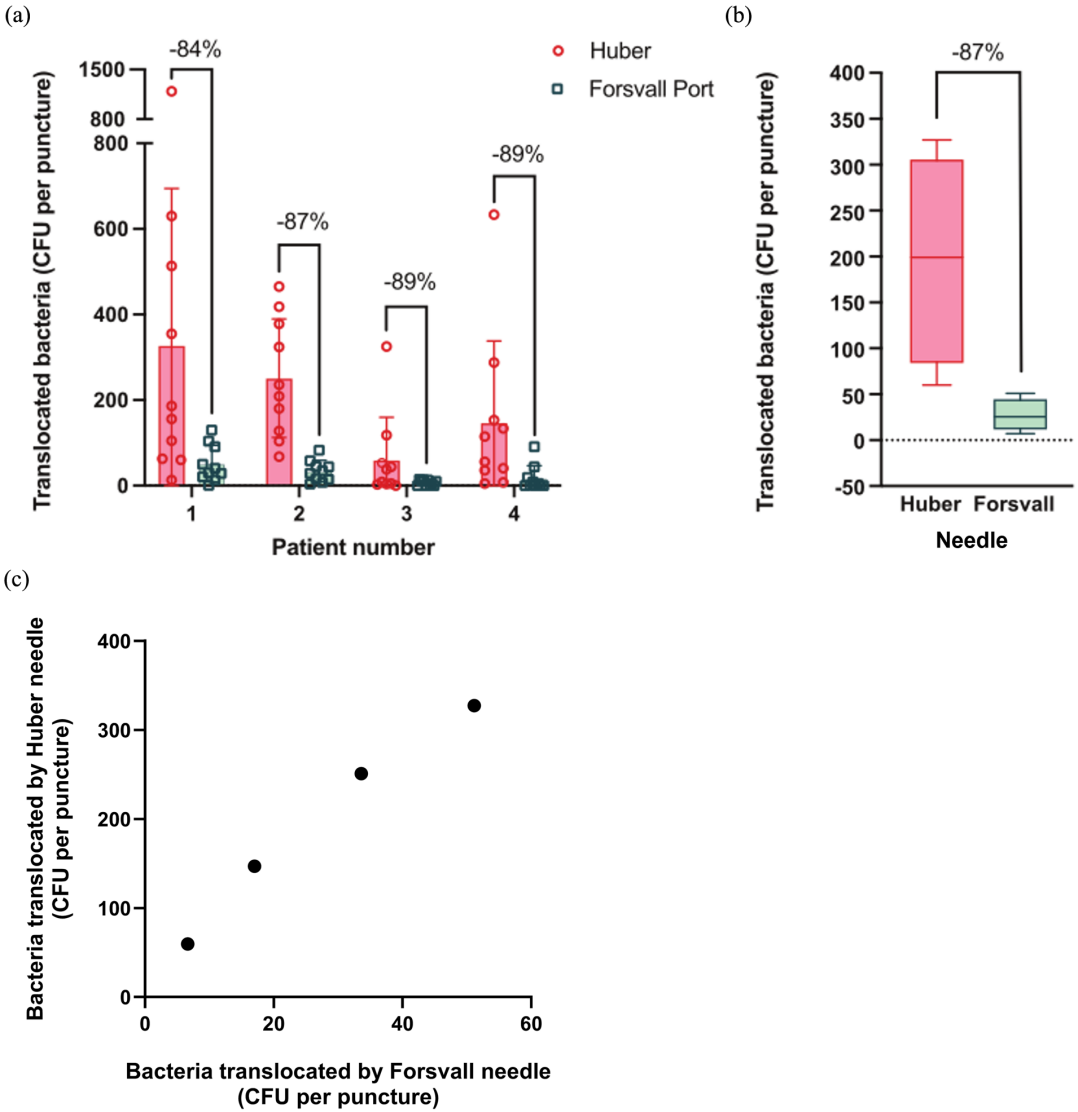
Bacterial translocation through ex vivo human skin per patient following 10 punctures with both the Huber and Forsvall Port needles. (a) The number of translocated bacteria per puncture for the Huber and Forsvall Port needles was measured after performing 10 punctures per skin sample from four different patients. Graph showing the percent reduction in translocated bacteria per puncture by the Forsvall Post needle relative to the Huber needle. Data points indicate the translocated bacteria for each puncture, bars represent the mean and whiskers are the standard deviation. (b) The pooled results (*n* = 40 for each needle type) from (a). Boxes represent the interquartile range, with the median shown as a line within the box. Whiskers indicate the range. (c) The average number of bacteria translocated by all punctures in each skin sample by the Huber needle was plotted against that of the Forsvall needle in the same skin sample.

To assess differences in bacterial translocation, we punctured *S. aureus*-coated skin from four healthy adults 10 times each per needle, eluted the bacteria with PBS, plated the eluates, and compared CFU counts (Figure 4). The Forsvall Port needle reduced the CFU per puncture by 84%–89% relative to the Huber needle (Figure 4(a)). On average, the Forsvall Port needle reduced the CFU per puncture by 87.0% (95% CI: 77.8%–92.4%, *p* < 0.0001, n_OBS_ = 80; Figure 4(b)). The appearance of the puncture holes was similar in both the skin and the TIVAP, with no evidence of TIVAP coring observed for either of the needles. The percent reduction in each skin was comparable despite the varying average CFU collected from the Huber needle (Figure 4(a) and (c)).

### Effect of needle tip closure on bacterial transfer across ex vivo skin

**Figure 5.**
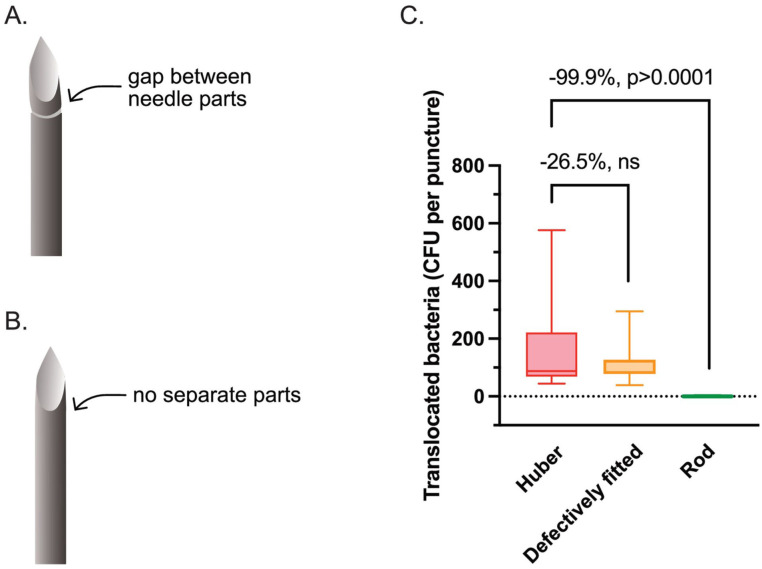
Substudy to evaluate the mechanism of bacterial transfer inside the Huber and Forsvall Port needles. A defectively fitted version of the Forsvall Port needle, with a gap where bacteria can enter inside the needle (a), or steel rod, where there is no entry point for bacteria (b), was punctured through the same ex vivo skin mounted on a TIVAP. (c) The number of translocated bacteria per puncture after performing 10 punctures per needle in skin from patient 5 with the Huber needle, defectively fitted Forsvall Port needle, and steel rod was measured. The box represents the interquartile range, with the median shown as a line within the box. Whiskers indicate the minimum and maximum values.

To test the hypothesis that bacteria are primarily transferred within the needle and tissue friction clears the outside of the needle, we examined in a fifth skin sample whether a solid steel rod (no opening) or a defectively fitted Forsvall Port needle (small gap between needle parts) could reduce bacterial transfer across the skin as effectively as a fully closed Forsvall Port needle (Figures 1 and 5(a) and (b)). The results were compared to the bacterial translocation resulting from puncturing the same skin sample with a Huber needle. We found that the steel rod reduced bacterial transfer by 99.9% (95% CI: 99.5%–100%, *p* < 0.0001, n_OBS_ = 20; Figure 5(c)), whereas the defectively fitted Forsvall Port needle resulted in a non-statistically significant 26.5% reduction in bacterial transfer (95% CI: 58.3% decrease to 30.0% increase, *p* = 0.289).

## Discussion

**Figure 6.**
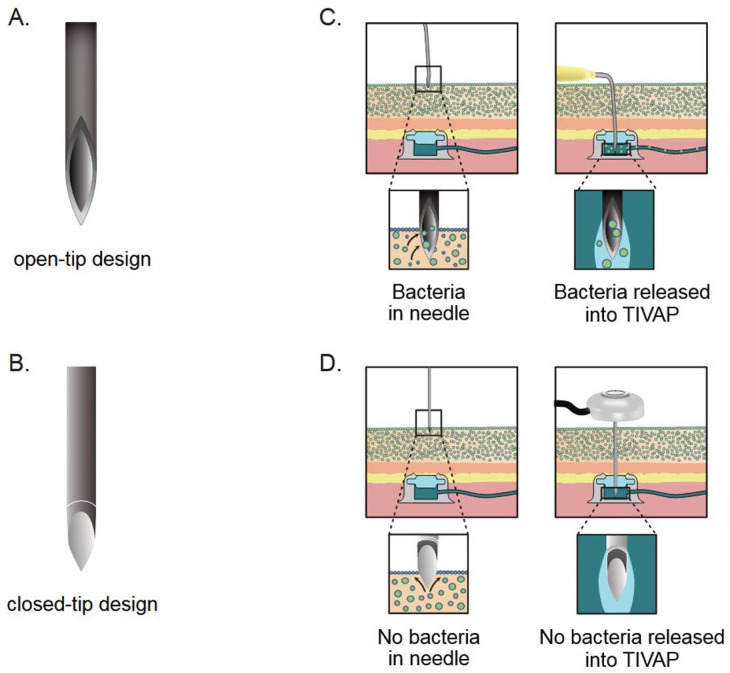
Design of the (a) Huber needle and (b) Forsvall Port needle used in this study. The Forsvall closed-tip needle design (b) significantly reduced bacterial transfer compared with the open-tip Huber needle (a). We hypothesize the results are due to the greater transportation of bacteria inside the Huber needle (c) than in the Forsvall needle (d) in punctures of TIVAPs. Bacteria on the outside of both needles are believed to be removed by tissue friction, but bacteria on the inside are protected from friction.

Our results show that a prototype novel needle with a closed-tip design (the Forsvall Port needle) reduces bacterial transfer from the skin into TIVAPs by on average 87% across 80 punctures compared with the currently used open-tip design Huber needle. This reduction in bacterial translocation could lower the risk of future infectious complications associated with TIVAP punctures and warrants further study in a clinical trial. We further propose that the Huber needle transfers more bacteria because microbes accumulate inside the open tip during skin penetration, where they are subsequently shielded from friction between the tissue and the needle as it moves through the skin and TIVAP (Figure 6(a) and (b)). Thus, tissue friction removes bacteria from the outside of both needles. A closed-tip needle design reduces bacterial transfer inside the needle and therefore significantly reduces microbial translocation across the skin (Figure 6(c) and (d)).

Infections associated with TIVAPs represent a significant global healthcare challenge, ranging from localized infections to severe systemic infections, such as septicemia.^7^ In oncology settings, infections might delay critical cancer treatment, requiring patients to undergo sepsis management, TIVAP removal, prolonged antibiotic therapy, and subsequent re-implantation of a new TIVAP.^19^ These complications not only increase patient morbidity but may also reduce a patient’s life expectancy and quality of life while placing a substantial burden on healthcare systems.^7^ Given that a TIVAP is an implanted foreign body lacking intrinsic immune defense, any reduction in bacterial transfer during needle insertion could theoretically lower infection rates, analogous to the current practice of skin sterilization prior to TIVAP access.^20–22^

Our results demonstrate a significant correlation between needle design and the extent of bacterial transfer. We found that the traditional open-tip Huber needle transfers significantly more bacteria across human skin compared with the closed-tip Forsvall Port needle. We propose that the difference is due to increased bacterial accumulation inside the Huber needle’s open tip (Figure 6). This theory is supported by observations that a defectively fitted Forsvall Port needle, which allowed bacteria to enter the needle lumen, failed to reduce bacterial transfer. In contrast, a solid steel rod simulating an optimally fitted Forsvall Port needle—effectively preventing bacterial entry—almost completely eliminated bacterial transfer (Figure 5(c)). Our findings also indicate that bacteria are almost exclusively transferred inside needles into TIVAPs, likely due to tissue friction removing bacteria from the outside of the needles during puncture. The findings of this study support the hypothesis that late TIVAP infections originate from bacteria transferred during the TIVAP-puncture procedure,^12,23^ specifically within the needle itself.

Bacteria may enter the TIVAP through injected fluids (via contaminated medications or connectors), directly through the needle, or as a secondary infection in cases of bacteremia.^24^ Current practice includes strict regulations in the sterility of fluids and medtech materials, skin surface sterilization, and comprehensive staff training programs. However, human error can occur, and surface sterilization does not penetrate the depths of the skin as the needle does.^15,25–28^ Studies suggest that even the most stringent preoperative skin preparation procedures leave up to 20%–25% of resident skin flora behind before surgery.^29–33^ Additionally, skin preparation before injections is often less stringent than for implantation procedures.

Furthermore, repeated needle insertions in the same small skin area may cause trauma of the skin covering the TIVAP. Traumatized skin and scar tissue is more prone to infection and the formation of biofilm.^34^ Biofilms produce an invisible extracellular polymeric substance matrix that acts as a physical barrier, shielding bacteria from antimicrobial agents and disinfectants. Bacteria in biofilms may enter a dormant state, making them less susceptible to surface sterilization methods.^35^ The combination of scar tissue and biofilm formation may further impede surface sterilization of the skin covering TIVAPs, allowing bacteria to persist and be collected by needles during penetration.^36^ This bacterial accumulation may partly explain the increased risk of infection associated with higher TIVAP usage.^12^ The use of a needle designed to prevent bacterial transfer, in conjunction with standard skin sterilization techniques, could represent a new strategy for reducing TIVAP-related infections.

This study has limitations to consider. The ex vivo study design used physiological concentrations of skin bacteria to simulate non-sterilized conditions, which may not fully reflect clinical scenarios where surface sterilization is routinely performed.

Although no patients were on antibiotics during the study, we did not register prior antibiotic use or establish any prestudy skin cultures. Despite our best efforts to administer a standardized amount of bacteria to the skins, there were differences in the transfer of total bacteria between skin samples, but our study was not designed to evaluate these differences. It is possible that these differences could be influenced by variability between skin samples with regard to diversity of skin microbiota, prior antibiotic exposure, skin thickness, and stretching upon mounting. Regardless, due to the study’s randomized, controlled design—wherein both needles were randomized across all skin samples—the final results are unlikely to be affected.

Consistent with a previous study in colon tissue,^13^ we observed a robust reduction in bacterial transfer across human skin when using a closed-tip needle. Again aligning with the earlier study, this reduction was independent of the bacterial load translocated by the Huber needle within individual skin samples, suggesting a consistent relative effect. The observed reduction in bacterial transfer (84%–89%) remained stable regardless of the total bacterial burden. This indicates that the effect may be independent of both the initial surface bacterial concentration and the sterilization technique employed, supporting the potential of the closed-tip design to complement existing skin disinfection protocols.

The Forsvall Port needle prototypes used in this study had imperfections, and could be further improved through manufacturing refinements, particularly in needle part alignment, which might further reduce the number of translocated bacteria. Based on physical properties, the Forsvall Port needle design is not anticipated to impact clinical usability or increase trauma to TIVAP septums,^37^ although this assumption needs to be thoroughly evaluated in future studies.

In the clinical setting, a needle is only used for one puncture. Due to a limited number of Forsvall Port needle prototypes available, each needle was used repeatedly in each skin sample. While needles may become duller with each puncture, we did not observe a trend toward increased CFU collection across repeated punctures.

With rising antibiotic resistance and a growing number of immunocompromised patients, it is crucial to develop strategies that prevent infections at their source. A novel needle design may provide a straightforward and effective approach to reducing the risk of infections in TIVAPs, ultimately improving patient outcomes and reducing healthcare costs. A randomized controlled trial is needed to assess the clinical impact of the Forsvall Port needle design on TIVAP infections.
